# Secure Fog Computing for Remote Health Monitoring with Data Prioritisation and AI-Based Anomaly Detection

**DOI:** 10.3390/s25237329

**Published:** 2025-12-02

**Authors:** Kiran Fahd, Sazia Parvin, Antony Di Serio, Sitalakshmi Venkatraman

**Affiliations:** Department of Business and Construction, Melbourne Polytechnic, Preston, VIC 3072, Australia; kiranfahd@melbournepolytechnic.edu.au (K.F.); saziaparvin@melbournepolytechnic.edu.au (S.P.); antonydiserio@melbournepolytechnic.edu.au (A.D.S.)

**Keywords:** fog computing, edge computing, AI-based anomaly detection, smart remote healthcare monitoring, intelligent data prioritisation

## Abstract

Smart remote health monitoring requires time-critical medical data of patients from IoT-enabled cyber–physical systems (CPSs) to be securely transmitted and analysed in real time for early interventions and personalised patient care. Existing cloud architectures are insufficient for smart health systems due to their inherent issues with latency, bandwidth, and privacy. Fog architectures using data storage closer to edge devices introduce challenges in data management, security, and privacy for effective monitoring of a patient’s sensitive and critical health data. These gaps found in the literature form the main research focus of this study. As an initial modest step to advance research further, we propose an innovative fog-based framework which is the first of its kind to integrate secure communication with intelligent data prioritisation (IDP) integrated into an AI-based enhanced Random Forest anomaly and threat detection model. Our experimental study to validate our model involves a simulated smart healthcare scenario with synthesised health data streams from distributed wearable devices. Features such as heart rate, SpO_2_, and breathing rate are dynamically prioritised using AI strategies and rule-based thresholds so that urgent health anomalies are transmitted securely in real time to support clinicians and medical experts for personalised early interventions. We establish a successful proof-of-concept implementation of our framework by achieving high predictive performance measures with an initial high score of 93.5% accuracy, 90.8% precision, 88.7% recall, and 89.7% F1-score.

## 1. Introduction

The rapid growth of the Internet of Things (IoT) [1], comprising interconnected devices embedded with sensors to collect, transmit, and exchange data, has introduced critical challenges in efficiency, security, and privacy in distributed environments [2,3]. Cyber–Physical Systems (CPSs), which integrate control, communication, and computational components with physical processes, form the foundation of IoT applications in domains such as smart cities, autonomous systems, and healthcare. These systems generate high-speed, heterogeneous, and time-sensitive data that require real-time analysis to support informed decision-making.

Traditional cloud computing has been the backbone of data storage and processing; however, its centralised nature is increasingly struggling with latency, bandwidth inefficiency, and privacy concerns in time-sensitive CPS applications. To mitigate these limitations, fog and edge computing have emerged as decentralised paradigms that extend computation and storage closer to data sources. Fog computing, in particular, offers low-latency and bandwidth-efficient processing but also raises new challenges, including resource-constrained data management, privacy preservation, and intrusion detection [4].

In the healthcare domain, especially for remote patient monitoring, these challenges are magnified. Continuous health data streams, such as heart rate, breathing rate, and oxygen saturation (SpO_2_), must be prioritised, secured, and analysed in real time to enable proactive clinical interventions. Existing fog-security frameworks have primarily focused on encryption and intrusion detection but have not adequately addressed the intelligent prioritisation of health data combined with AI-based anomaly detection, which is crucial for timely and reliable decision-making.

To address these limitations, this study introduces a novel fog-based framework that combines multiple complementary components. First, an Intelligent Data Prioritisation (IDP) mechanism ranks and filters critical health data streams based on medically defined thresholds and contextual urgency, ensuring that life-critical information is processed without delay. Second, a Random Forest-based anomaly detection model is integrated to identify clinically significant anomalies that may not be captured by static threshold rules, thereby enhancing the early detection of potential health risks. Finally, the framework incorporates privacy-preserving, lightweight security mechanisms, including secure transmission channels and scalable encryption, to safeguard sensitive health data in resource-constrained fog environments.

These contributions collectively advance beyond traditional fog-security frameworks to a prioritisation and intelligence-driven fog architecture tailored for healthcare CPS. Although demonstrated here in a smart healthcare scenario, the proposed approach is extensible to other CPS domains, such as smart cities, industrial automation, and intelligent transportation systems. Overall, the key features of this paper are summarised below:

- The focus of our research problem is to propose a novel fog architecture integrating IDP, AI-based anomaly detection and dynamic data prioritisation.
- The scientific methods adopted aim to address the drawbacks of existing frameworks and to alleviate the gaps found in the literature. The enhanced Random Forest method used for AI-based anomaly detection adopts an ensemble of decision trees to improve accuracy and robustness.
- Our main contribution lies in the development of a unique framework for secure fog computing with IDP and AI strategies in an enhanced Random Forest for anomaly detection. Our innovative AI-based model incorporates medical context-dependent expert rule based data prioritisation in a dynamic and distributed environment with experimental validations performed in a stimulated healthcare scenario.

The rest of the paper is organised as follows. Section 2 provides background and related work of the study. Section 3 details our proposed secure fog computing framework and its components. In Section 4, we demonstrate the application of our framework by experimenting with remote patient simulation monitoring using sensitive medical datasets, showcasing the promising results. Section 5 provides discussions and recommendations derived from this study. Finally, Section 6 provides conclusions and future research directions.

## 2. Background

The exponential growth in data generated by IoT-enabled CPS devices, combined with the complex technologies of autonomous cars that utilise radar, cameras, sensors, and AI, has overwhelmed cloud architecture. Traditional cloud architecture is unable to manage latency and performance issues created by the distance between centralised private and public cloud data centres and end-user devices. To overcome these limitations, edge and fog computing paradigms emerged, enabling low-latency, localised processing closer to the CPS data source.

### 2.1. Related Work

Several efforts in the literature have proposed using fog computing for industrial and CPS-related applications, such as [5,6]. Other efforts addressed energy efficiency and resource management in CPS using fog computing in [7,8,9]. Existing literature has primarily addressed fog-based frameworks for IoT-enabled CPS applications, with emphasis on either security, latency optimisation or anomaly detection in isolation. However, limited work has focused on CPS devices in health settings that integrate IDP with AI-driven anomaly detection.

Recent research in fog IoT environments has focused on securing the environment. For example, the work in [10] proposed a zero-trust framework integrated with blockchain and SDN to secure fog networks and highlighted the utilisation of cryptographic protocols and access control. Another work [11] surveyed approaches such as lightweight encryption, intrusion detection, and privacy-preserving measures for fog-based health applications. Similarly, ref. [12] also reviewed the associated security challenges in fog environments, including access control and threat mitigation. Although these works help secure fog environments, they primarily overlook IDP and AI-driven anomaly detection, which are essential for real-time healthcare monitoring. We find that Random Forest algorithm consistently ranks among the top performers in anomaly detection, particularly in healthcare and security contexts. Hence, our model leverages a Random Forest ensemble of decision trees to enhance both accuracy and robustness compared to single-model approaches. We construct multiple decision trees using randomised feature subsets of health data and then aggregate their votes to determine if a data point is anomalous, which improves predictive reliability.

Furthermore, multiple existing studies have addressed data prioritisation for IoT in healthcare settings. For example, a study [13] introduced a computer vision-based IoT architecture that used predefined thresholds and classified health data as high or low priority. Another work [14] presents a comprehensive IoT-based architecture for smart hospitals that integrates clinical information systems (CIS) to improve interoperability, security, and overall healthcare efficiency. Similarly, another study [15] was conducted on dynamic priority-based task scheduling and resource allocation for optimising IoT-based resource usage and scheduling in healthcare.

The primary focus of this prioritisation was tasks rather than anomaly detection. Recent research [16] explored real-time anomaly detection using wearable devices to learn personalised baselines for detecting irregularities. A study [17] employed unsupervised models to identify anomalies in digital health data, while another study [18] utilised deep learning to develop a cloud-based framework for detecting ECG anomalies. These studies highlight the importance of prioritisation or demonstrated strong predictive capabilities but are still limited in their ability to capture significant deviations in healthcare data in real time or to operate in a cloud environment.

Existing research focuses on security aspects; healthcare IoT initiatives often prioritise data using static thresholds; and AI-driven anomaly detection remains in the cloud. Thus, the existing literature lacks an integrated approach that combines secure fog computing with IDP and AI-driven anomaly detection tailored for remote health monitoring. This study addresses this gap by proposing and evaluating our unique framework integrating IDP, security mechanisms and AI based anomaly detection over Fog computing environment for remote healthcare settings.

### 2.2. Fog and Edge Computing Environments

#### 2.2.1. Edge and Fog Computing

Edge computing is a method used to reduce the transmitted data load and minimise latency between data collection and processing, which occurs directly on the device with the capacity (sensors attached or a gateway device physically close to the sensors) to process its data locally. For instance, imagine a device that continuously captures data using a sensor, sends the data, with minimal processing or encryption, as-is” to a centralised cloud network (typically for storage and analysis). The cloud is considered the “centre”. In contrast, the smart device is on the “edge” of the network. Fog computing is defined as the standard of how Edge computing should work, which was coined by Cisco Systems in 2012 [19]. As shown in Table 1, fog computing acts as a bridge between the edge and cloud layers by providing near real-time analytics, localised security, and moderate scalability [20,21,22,23].

**Figure 1:**
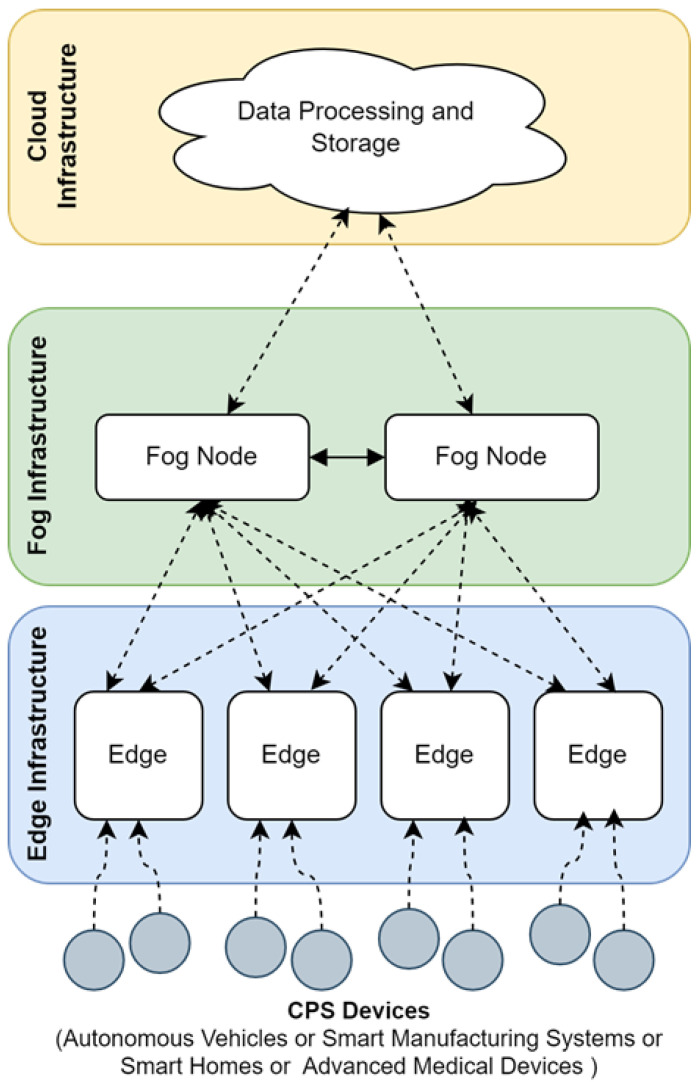
Cloud, Fog, and Edge computing architecture.

In the current era of technology, most applications require real-time data exchange. As a result, it requires a massive data warehouse (big data) to store the data. Only the relevant data is sent to the cloud for processing and storage, which maximises the efficiency of the cloud platform. In fog computing, latency is reduced by utilising multiple fog servers, which provides sufficient space for each fog server to store data. The increased data volume requires databases with greater availability and scalability to address the issue. Fog computing provides a data service layer with non-relational databases, offering solutions that support scalability, flexibility, high availability, low latency, schema-less design, and the handling of unstructured data [24,25]. Non-relational databases are designed and developed to handle such situations and are the best alternative to SQL databases. Data can be maintained in a distributed manner using NoSQL databases, and the performance of non-relational databases enables real-time data processing in Fog computing [26]. For example, Apache Cassandra is a great database solution used in fog servers on the Internet edge, offering high scalability and a distributed system that enables the management of streaming data in CPS environments. Figure 1 shows the cloud, fog, and edge computing with CPS devices.

#### 2.2.2. Edge and Fog Paradigm and Smart Healthcare

The above explanation of fog computing highlights that fog computing is well-suited for healthcare applications where timely responses, privacy preservation, and reduced network dependency are critical. Therefore, the proposed framework leverages fog computing to achieve secure, efficient, and intelligent data processing for remote health monitoring. Recently, IoT-enabled CPS devices have been widely adopted in healthcare to enable continuous monitoring of patient vital signs and improve patient outcomes using smart wearables and sensors, such as smart watches or Smart Biosensor Patches. These devices monitor health metrics such as heart rate, sleep patterns, physical activity, oxygen levels, stress levels, or glucose levels and generate real-time heterogeneous data that utilises a cloud-based architecture [27,28]. These cloud-centric infrastructures face limitations and constraints that highlight privacy risks, especially in time-sensitive health-related scenarios. The layered architecture comprises IoT-enabled CPS devices, edge and fog layers, and cloud infrastructure, thereby enhancing data privacy and responsiveness [29]. Also, this layered architecture has proved effective in smart healthcare monitoring cases [30]. These advancements underscore the role of cloud computing in improving healthcare delivery, particularly by enabling secure, real-time patient care.

#### 2.2.3. Security Challenges of Fog Computing

Fog computing provides data processing and storage services to end users, similar to cloud computing [31]. The existing literature identifies security challenges related to fog computing, including authentication, data protection, and privacy [3]. Fog computing provides services to many users through fog nodes. Without proper authentication, messages and entities will expose a significant risk of compromise, potentially leading to a man-in-the-middle attack [31,32]. However, implementing face, biometric, and touch-based authentication helps prevent authentication issues in fog computing.

Due to the increased number of CPS devices and the data they generate, it is incredibly challenging to handle the large volumes of data sent to the nearby fog node. Since it is difficult to process substantial amounts of data on CPS devices, the data is divided and sent to multiple fog nodes for processing. Data integrity should be ensured in the distributed data, but due to resource constraints, the encryption and decryption of data are challenging [32]. Hence, lighter encryption and masking techniques should be implemented to address the issue.

Privacy is the most challenging aspect to address in fog computing, as fog servers are located very close to end users and handle extremely sensitive data, unlike in cloud computing. The privacy of location, data, and services must be protected, as each fog node exchanges sensitive information with others. Hence, the location of the data should be protected from each fog node to establish and provide reliable communication [33]. Threats and DDoS attacks disrupt the production process and cause communication issues with IoT services and applications. Therefore, intelligent fog computing can provide countermeasures against DDoS attacks and strategies to mitigate such attacks at scale [34].

## 3. Proposed Framework

**Figure 2:**
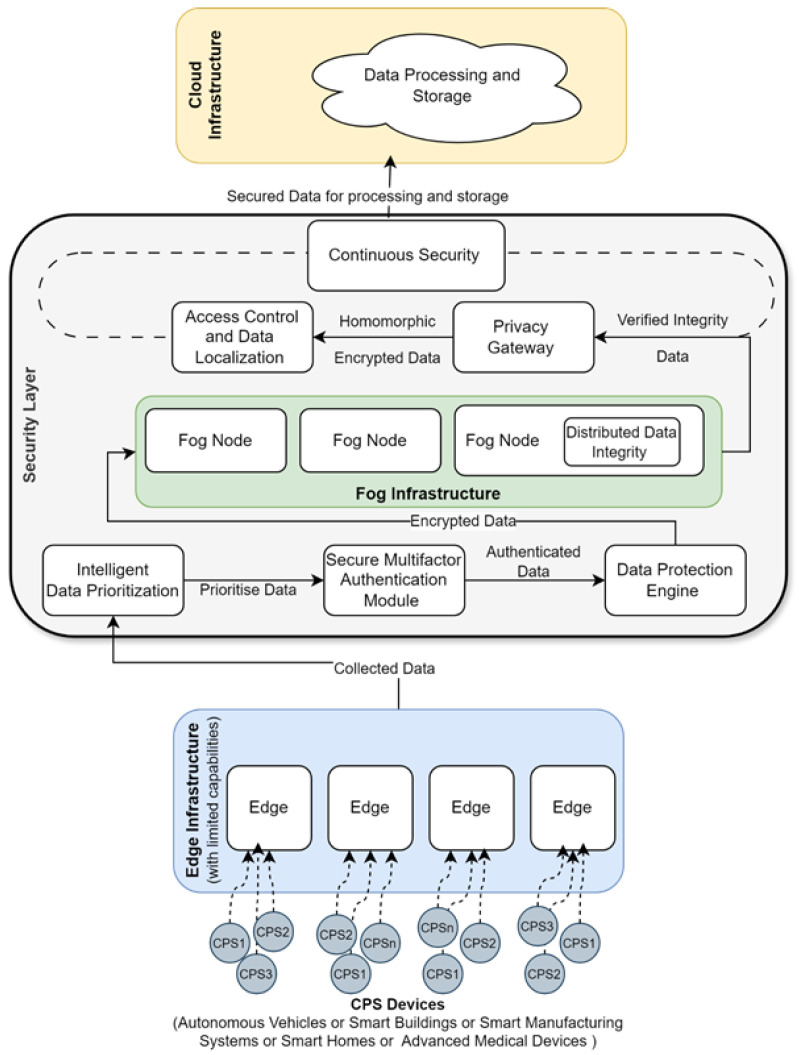
Proposed secure fog computing framework integrating IDP and AI anomaly detection.

This study proposes a secure fog computing framework tailored for remote health monitoring that integrates three key capabilities: IDP, lightweight security mechanisms, and AI-based anomaly detection. The framework addresses the critical challenges of authentication, privacy, and efficient handling of massive health data streams from IoT-enabled CPS devices by processing information closer to the source while maintaining strong safeguards. Figure 2 illustrates the layered architecture and interactions between its components, including end-to-end secure transmission from edge devices to fog nodes.

Unlike conventional fog or cloud solutions that treat security, prioritisation, and anomaly detection as separate processes, this framework unifies these capabilities into a single workflow. This integration ensures that health data are prioritised in real time, transmitted securely, and analysed for anomalies at the fog layer, enabling timely clinical responses and reduced reliance on central cloud systems.

The workflow begins at the edge layer, where health data streams (e.g., heart rate, SpO_2_, and breathing metrics) are collected. Before entering and leaving the edge layer, data are encrypted using lightweight cryptographic protocols for secure transmission to the fog. An IDP Engine applies threshold-driven and AI-enhanced ranking to ensure that clinically urgent data are transmitted first, while routine or noncritical information is deferred for later processing. This reduces bandwidth consumption and provides rapid delivery of high-priority signals to caregivers.

Once prioritised, the data undergoes authentication and encryption. Multi-factor authentication mechanisms validate devices and users to prevent unauthorised access, while lightweight encryption ensures data confidentiality both at rest and in transit. These measures provide a strong security foundation under the resource constraints typical of fog deployments. At the fog nodes, the data is decrypted, integrity-checked, and stored in a distributed manner that prevents tampering or corruption. Additional safeguards, such as privacy-preserving gateways, can support encrypted data analysis and enforce data localisation policies, thereby addressing compliance and regulatory requirements. Finally, an AI-based anomaly detection module operates directly at the fog layer. A Random Forest model analyses prioritised health data streams to identify anomalies that static threshold rules may not capture. By detecting early signs of potential health deterioration, this component enhances proactive patient monitoring and intervention. The existing literature [35,36,37] shows that the Random Forest algorithm consistently ranks among the top performers in anomaly detection, particularly in healthcare and security contexts. Our proposed framework leverages a Random Forest ensemble of decision trees to enhance both accuracy and robustness compared to single-model approaches. Random Forest constructs multiple decision trees using randomised feature subsets and then aggregates their votes to determine if a data point is anomalous, which improves predictive reliability. In our proposed framework, we have enhanced the Random Forest method in a unique way by integrating intelligent data prioritisation of distributed clinical data modelled by dynamic medical threshold rules in a fog environment. Further, the high-priority health data streams stored in Cassandra are continuously observed by machine learning models trained to detect personalised abnormal patterns in patient health metrics and unusual access behaviours in the system. These key enhancements highlight the RF model’s betterment. By integrating secure transmission, intelligent prioritisation, and AI-based anomaly detection, the proposed framework offers a reliable, scalable, privacy-preserving, and efficient approach to remote health monitoring in fog-based environments.

## 4. Experimental Design and Evaluation

This section illustrates the application of the proposed secure fog computing framework in a remote health monitoring setting. Such systems link wearable and implantable medical devices, such as cardiac sensors, glucose monitors, and neurostimulators, to continuously gather and transmit patient health information. While these technologies facilitate the proactive management of chronic illnesses, such as cardiovascular disease and diabetes, they also pose challenges related to data privacy and security, as well as the efficient handling of large data streams. By incorporating secure data transmission, IDP, and AI-based anomaly detection, the framework enhances data security, ensures critical signals are prioritised, and supports prompt medical responses by healthcare providers.

### 4.1. Simulation Setup for Remote Patient Monitoring with AI Anomaly and Threat Detection

To validate the practicality of the proposed framework, a prototype was tested in a simulated setup for smart remote health monitoring with AI-based anomaly and threat detection. A synthetic dataset was created to replicate real-time data from wearable devices, such as Fitbit sensors, while references to commercial devices ensured realistic simulation conditions for anomaly and threat detection. The framework continuously collects health metrics from these simulated edge devices, where the IDP module ranks and transmits urgent signals, such as abnormal heart rates or oxygen saturations, directly to the fog layer for immediate analysis. AI-based anomaly detection is then performed at fog nodes to identify potential clinical risks beyond static thresholds. Although no live devices were used during this phase, the simulation demonstrates the framework’s feasibility and reliability for managing, prioritising, and securing health data in real time. Instead, patient health data, such as heart rate, SpO_2_, and breathing rate, were synthetically created to simulate continuous monitoring over one month, i.e., 10,000 simulated patient data points. The dataset features include device ID, timestamp, heart rate, heart rate priority, SpO_2_, breathing rate, SpO_2_ priority, and breathing rate priority. The data is collected periodically, every hour, for 5 Fitbits over a month. Table 2 provides details of the dataset, designed to simulate realistic critical health monitoring data streams and to demonstrate both data prioritisation and AI-driven anomaly detection. The Status column is marked as ‘alert’ if the heart rate exceeds 100, and ‘ok’ otherwise.

This experiment has simulated the use of multiple Fitbits to record and monitor heart rate, oxygen saturation (SpO_2_) (measures the oxygen level in the blood) and breathing rate of a remote patient and collect data over one month. Heart rate, oxygen saturation, and breathing rate metrics are critical indicators of a patient’s heart health, respiratory function, and overall well-being. Kafka ingested the simulated Fitbit data streams, applied predefined health thresholds in the IDP to intelligently prioritise urgent patient data, and stored the high-priority data in Cassandra for secure storage and retrieval using AI-based anomaly and threat detection.

Multiple APIs were programmed to simulate periodic checks of heart rate, SpO_2_, and Breathing Rate every hour. All health data generated by Fitbit is securely transmitted to fog nodes using encrypted communication channels, i.e., HTTPS. At the fog nodes, Kafka manages the data streams coming from the edge nodes. The Kafka stream engine intelligently prioritises incoming health rate data based on its processing and storage urgency, comparing each metric against expert knowledge based thresholds personalised for patients. For example, flagging abnormal heart rates or oxygen levels, i.e., heart rates above 180 bpm [38], SpO_2_ below 90% [39], or breathing rate less than 12 or greater than 20 breaths per minute [40], as high-priority.

In this experiment, the collected prioritised timestamped data about device ID, heart rate, SpO_2_, breathing rate, and respective priority classifications are simulated to assess the framework’s ability to identify critical conditions, such as respiratory distress or cardiac anomalies, using real-world medical criteria. All these attributes collectively support anomaly detection by providing both clinical indicators and contextual features. At the same time, the Status attribute acts as a classification label for training and validating AI models designed for detecting anomalies and threats. The simulation environment generated data representing the heart rate from the Fitbits. The heart rate data includes both normal and abnormal heart rates to evaluate the intelligent prioritisation mechanism at the fog node, with values ranging from 60 to 180 bpm. Similarly, the proposed framework prioritised health data as high-priority if the SpO_2_ level was below 90% or the breathing rate was either above 20 breaths per minute or below 12 breaths per minute. Predefined thresholds help identify critical health conditions in remote patients, such as heart disease, potential sleep apnoea, or respiratory distress. The results showed that the proposed framework effectively filtered and prioritised life-critical health events in a secure environment for prompt intervention and identified health issues needing immediate attention.

### 4.2. AI-Driven Anomaly and Threat Detection

In addition to data prioritisation and secure transmission, the framework incorporates AI algorithms to identify anomalies within the smart remote patient monitoring system. The high-priority health data streams stored in Cassandra were continuously observed by machine learning models trained to detect abnormal patterns in patient health metrics and unusual access behaviours in the system. For clinical anomalies, supervised models compare incoming data against medically accepted ranges to detect conditions such as sudden cardiac irregularities or respiratory distress. For instance, deviations in normal heart rate variability or trends in oxygen saturation are flagged as potential health risks requiring immediate attention. During preprocessing, the continuous characteristics (Heart Rate, HR, SpO_2_, Skin Temperature) were normalised using the Min–Max scaling method as shown below:

X’=x−min(x)(max(x)−min(x))

In the above formula, X′ is an individual value of each attribute, and min(x) and max(x) denote the minimum and maximum values of that attribute in the dataset. Normalisation scales all features to the same range, so each feature contributes to model training in proportion to its range.

**Figure 3:**
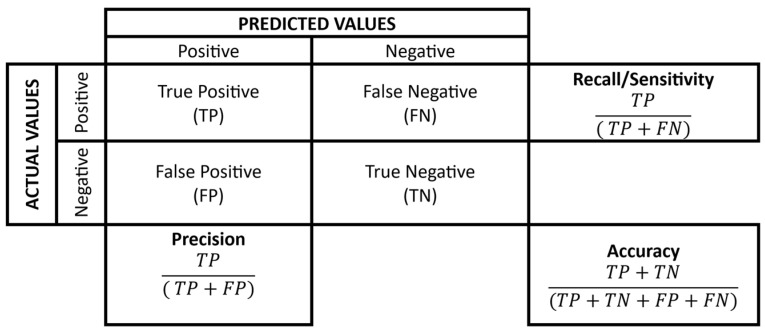
Performance metrics.

The ‘Exercising’ feature is encoded as 0 and 1. This study uses a Random Forest classifier, an ensemble learning method, on a diverse clinical dataset to test its robustness. The parameters set were: 200 trees and a maximum depth of 10. Relevant features are identified and ranked using the Random Forest feature importance technique. A 10-fold cross-validation was employed, splitting the dataset into 10 equal, random subsets. In each iteration, nine subsets are used for training, and the remaining one for testing. The results are averaged across all 10 runs to produce the final outcome. Feature importance is calculated during cross-validation. To assess the framework’s ability to detect clinical anomalies, metrics such as classification accuracy, precision, recall, and F1-score are used. Accuracy shows the percentage of correct predictions overall. Precision indicates the proportion of predicted anomalies that are true, reducing false alarms. Recall measures the percentage of actual anomalies correctly identified, minimising missed critical events. The F1-score combines precision and recall, offering a balanced performance measure. The confusion matrix, as shown in Figure 3, is a performance measurement for ML models. Break down the prediction of the model into true positives (TP), false positives (FP), true negatives (TN), and false negatives (FN), and provide the calculations for several key metrics.

### 4.3. Results

To assess the efficacy of the proposed framework, a simulated environment was established to exemplify a real-world smart remote patient monitoring system using synthetically generated Fitbit data. Data streams for different health metrics, such as heart rate, SpO_2_, and breathing rate, were produced for five simulated edge devices (Fitbit) over one month. Libraries such as Seaborn (0.13.2), Matplotlib (3.8.2), and Scikit-learn (1.4.2) from the Python ecosystem were used to visualise and analyse the prioritisation and classification of anomalies in vital health events at the fog layer.

**Figure 4:**
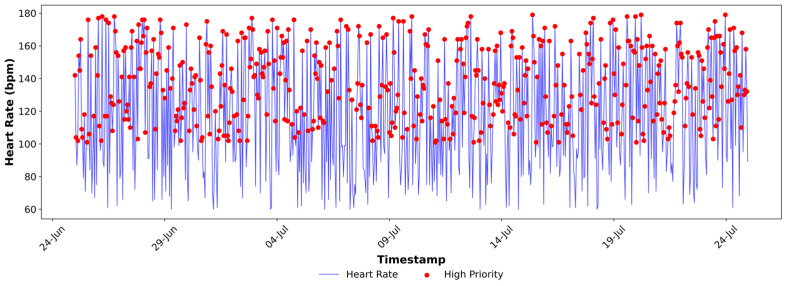
High-priority Indication of the Heart Rate Data at Fog Node.

**Figure 5:**
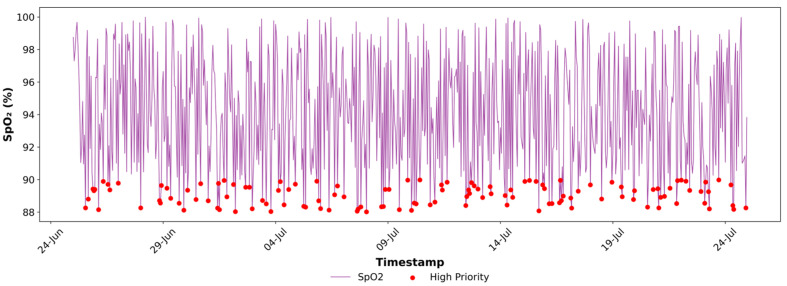
High-priority Indication of the SpO_2_ Data at Fog Node.

**Figure 6:**
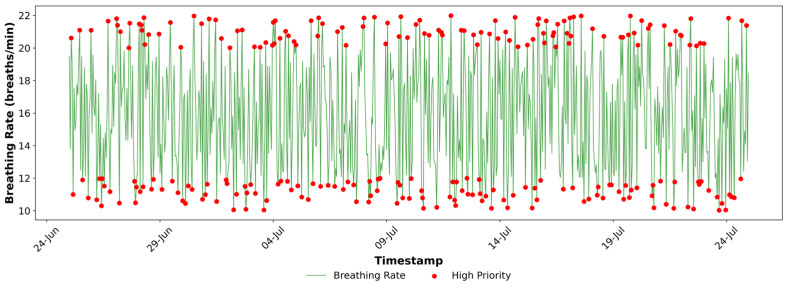
High-priority Indication of the Breathing Rate Data at Fog Node.

**Figure 7:**
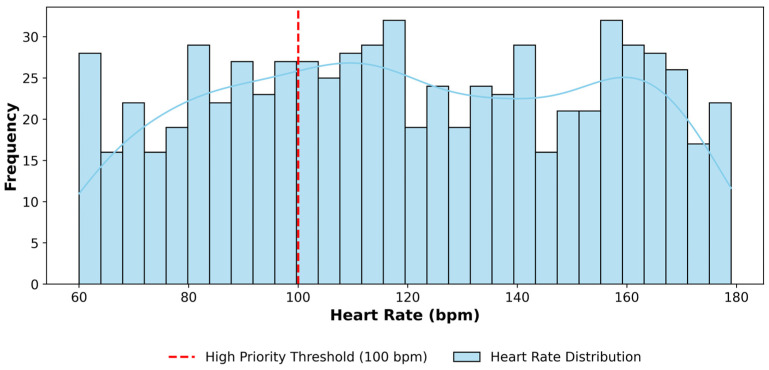
Distribution of High-priority Heart Rate Data at Fog Node.

**Figure 8:**
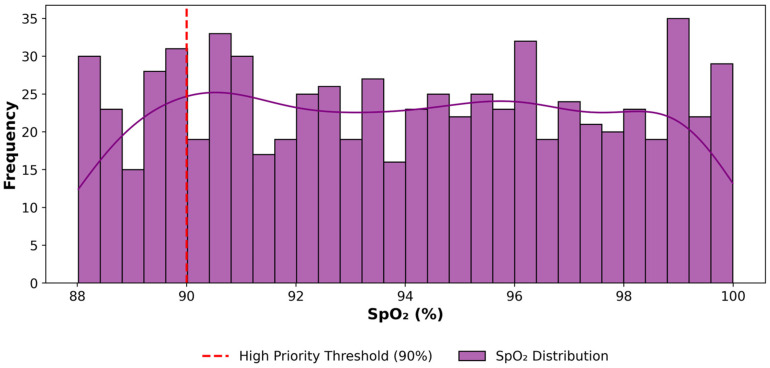
Distribution of High-priority SpO_2_ Data at Fog Node.

**Figure 9:**
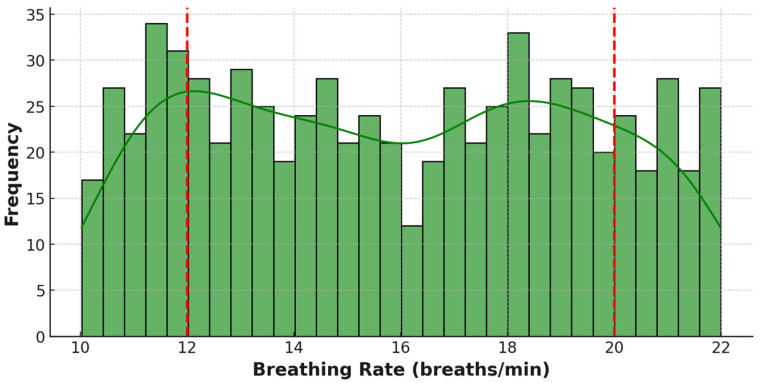
Distribution of High-priority Breathing Rate Data at Fog Node.

Figure 4, Figure 5, Figure 6, Figure 7, Figure 8 and Figure 9 provide a clear, informative visualisation of the simulated experiment to analyse heart rate, SpO_2_, and breathing rate measurements and prioritise critical health data for a single device. Figure 4 presents the hourly heart rate data from a single simulated device (Fitbit) out of five devices over a month. Critical readings exceeding 100 bpm, which are considered high-priority, are highlighted in red. These anomalies may correspond to times of stress or other health conditions, which can cause an increased heart rate. The visual graph identifies an instance of abnormal heart rate, which is crucial for monitoring health and might indicate a condition that requires attention, such as Arrhythmias.

Similarly, Figure 5 displays the prioritisation of SpO_2_ data by plotting SpO_2_ readings within the normal range and indicating high-priority readings with red markers, denoting drops below 90% and potentially signalling hypoxemia or early signs of respiratory compromise.

Figure 6 illustrates the breathing rate readings and the high-priority flags (in red), which identify values outside the normal range of 12–20 breaths per minute. These instances of abnormal breathing rates over one month indicate issues with respiratory health. They may serve as a symptom of an underlying respiratory disorder, such as hyperventilation or other respiratory irregularities.

Figure 7 illustrates the statistical distribution of heart rate over one month, providing insight into heart rate readings. The vertical line indicates the high-priority threshold. It helps identify common and extreme values in heart rate data and understand the distribution of heart rate levels. The blue trend curve highlights the overall pattern of heart rate readings across the month, showing how the distribution shifts around the high-priority threshold. 

Similarly, Figure 8 shows the frequency distribution of all SpO_2_ readings, with the red vertical line indicating the high-priority threshold of 90% oxygen level across the entire simulation period. This instance represents a typical instance, indicating normal oxygen levels for the majority of the time over one month. The purple trend curve shows the overall trend of SpO_2_ readings across the month, demonstrating how the distribution aligns with the 90% threshold.

The statistical distribution of breathing rate measurements across the whole simulation period at the fog node is shown in Figure 9. Most of the readings fall within the normal breathing rate range of 12–20 breaths per minute. However, there are a few notable readings outside the thresholds, lower than 12 breaths per minute or higher than 20 breaths per minute, indicating potential issues with the patient’s respiratory system. The green curve highlights the overall pattern of breathing rate occurrences and shows how the distribution corresponds with the normal 12–20 breaths per minute range. 

Figure 7, Figure 8 and Figure 9 include a threshold marker that delineates high-priority boundaries for prompt pattern recognition by healthcare providers or monitoring systems. Therefore, this data can be used to monitor human health over time and identify patterns and trends in critical health metrics such as heart rate, SpO_2_, and breathing rate, enabling proactive health management. This high-priority data not only helps identify how often these events occur but could also be used to trigger alerts that notify healthcare providers of potential health issues. In total, the framework flagged 182 high-priority events across all three metrics, validating its ability to automatically identify and classify vital health data for timely care escalation in a smart remote healthcare scenario.

**Figure 10:**
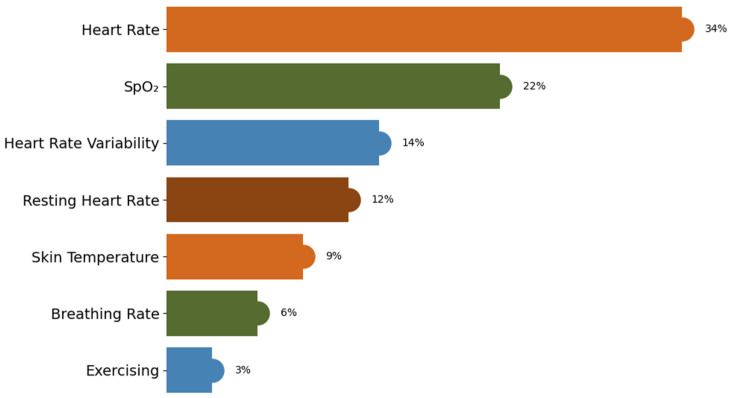
Feature Importance Plot using Matplotlib.

Building on this data prioritisation, the proposed framework is further evaluated using a Random Forest classifier to assess its ability to detect anomalies within the prioritised health data. This enables the detection of anomalies that may not be captured by using the threshold only. The Random Forest-based model is tested using k-fold cross-validation (where k = 10). Feature importance analysis is performed to determine which attributes of the health data contributed most to anomaly classification. Features such as heart rate and SpO_2_ are the dominant predictors of anomaly detection. Other features, such as heart rate variability, resting heart rate, and skin temperature, also contributed meaningfully. Figure 10 illustrates that all seven selected features have non-zero importance values, indicating that each feature contributes to predicting both normal and anomalous health states. The Python Matplotlib data visualisation library is used to demonstrate the relative importance of each dataset feature.

**Figure 11:**
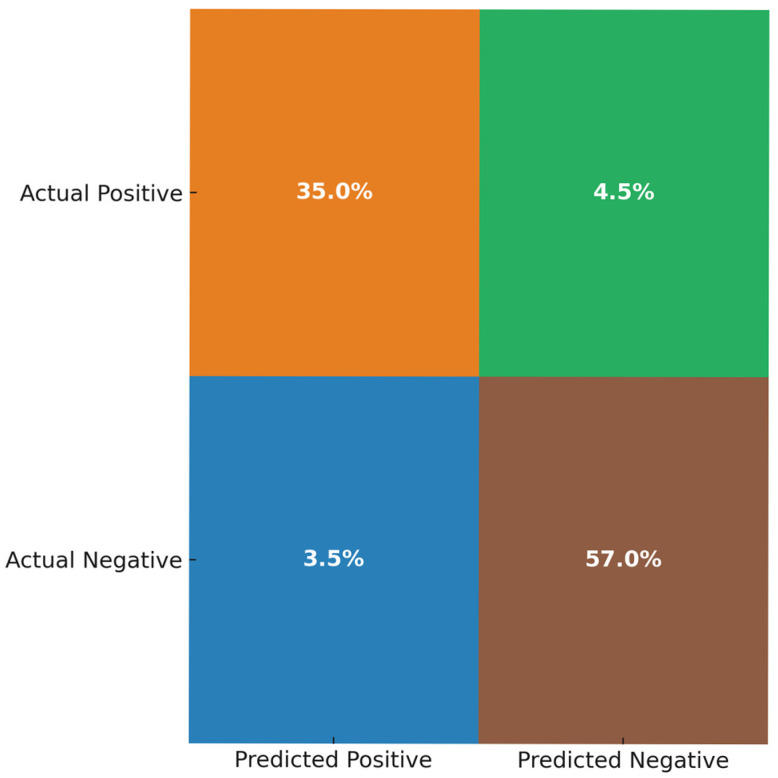
Confusion Matrix for Random Forest based Model for anomaly detection.

**Figure 12:**
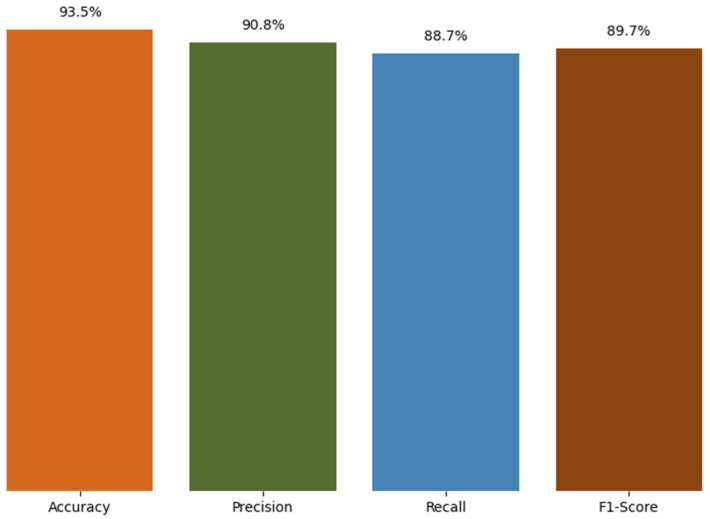
Model performance metrics based on the Random Forest algorithm for anomaly detection.

In this study, Random Forest, an ensemble-based ML algorithm, is applied to the dataset to train and test a predictive model for anomaly detection to improve reliability. The model’s performance is evaluated using a confusion matrix, which highlights the distribution of TP, FP, TN, and FN, from which the evaluation metrics of accuracy, precision, recall, and F1-score were derived, as depicted in Figure 11 and Figure 12. 

The key performance metrics of the model include accuracy, precision, recall, and F1-score, which provide a comprehensive assessment of how effectively it identifies both positive and negative instances. The accuracy, precision, recall, and F1-score are calculated as given in Table 3.

In the context of this study, anomaly detection refers to the identification of health data that deviates from clinically accepted norms or set thresholds, such as elevated heart rates or low SpO_2_ levels (e.g., below 90%). Such critical data may signal early indications of health issues and thus require timely intervention. The model achieved strong predictive performance in detecting anomalies within the prioritised health data. 93.5% of the data points were correctly classified. A 90.8% precision rate indicates that the majority of anomaly alerts generated by the trained model correspond to true anomalies, thereby mitigating false alarms. Furthermore, an 88.7% recall highlights the ability to capture most of the actual anomalies, thereby minimising the risk of missing critical health alerts. The combination of high precision and strong recall ensures that the model’s anomaly detection is both clinically meaningful and comprehensive in identifying potential health risks.

This simulated experiment shows how fog nodes and edge computing with smart medical devices can improve latency, secure data processing, and manage sensitive health information. Although tested in a controlled environment using simulated data, it indicates potential advantages for practical use, including scalable alerts, bandwidth savings, enhanced security, and real-time monitoring. These benefits can be summarised as follows:

- Processing fog node data can reduce latency and enable near real-time insights into patient health, as shown by anomaly detection in the simulation.
- Preliminary fog analysis can detect potential health problems before cloud processing, decreasing unnecessary data transfer and prioritising urgent information.
- Fog nodes can be set up to activate automated alerts whenever thresholds are exceeded, allowing timely intervention.
- Implementing local filtering and processing at fog nodes can decrease the amount of data transmitted to the cloud, enhancing bandwidth efficiency.
- Processing nearer to the source can reduce security risks during transmission.
- The anomaly detection model based on Random Forests showed reliable results across various dataset partitions.
- The ensemble approach of the Random Forest model minimises overfitting risk and enhances its ability to generalise.
- The Random Forest model demonstrated high accuracy in detecting anomalies, showcasing its reliability for identifying critical health data irregularities.

Overall, these results demonstrate the proof-of-concept viability of the proposed framework as a secure, scalable, and efficient method for IoT-enabled CPS to monitor and manage patient health at the edge. Also, these results should be viewed as proof of concept, as simulation-based validation with real-world datasets remains to be conducted.

## 5. Discussion and Recommendations

This study highlights that combining secure fog computing with IDP and AI-driven anomaly detection offers a scalable, dependable alternative to traditional cloud-centric methods. Instead of emphasising only the overall evolution of cloud technologies, the study demonstrates that adding layered security, prioritisation, and anomaly detection at the fog layer directly addresses issues of latency, bandwidth, and privacy in centralised cloud processing. For applications such as remote healthcare monitoring and other CPS tasks, a hybrid system that effectively integrates cloud, fog, and edge computing is most beneficial. The fog layer not only enhances security but also enables real-time prioritisation and intelligent anomaly detection for critical health data streams. A key contribution of this study is the integration of anomaly and threat detection with prioritised health data streams. While IDP guarantees that crucial health data is escalated in real time, the following Random Forest-based model builds on this by learning subtle patterns that may not be caught by threshold-based rules only. In our experiments, the Random Forest model achieved high accuracy (93.5%), precision (90.8%), recall (88.7%), and F1-score (89.7%), confirming its effectiveness for anomaly detection in prioritised health data. Also, the Random Forest-based model offers a practical alternative for fog-based environments, as it combines strong predictive performance without the high computational demands. Integrating prioritisation with anomaly detection in health settings provides a control mechanism to reduce FP and prevent FN delays in critical interventions. However, these findings should be regarded as preliminary with simulation-based validation and testing with real datasets has been earmarked as future work.

### 5.1. Summary of Our Research Contribution

As with any distributed system, fog computing presents security and privacy challenges, underscoring the inadequacy of traditional cloud security approaches. This simulated experiment demonstrates the feasibility of deploying fog nodes and edge computing with smart medical devices to enhance latency, secure processing, and management of sensitive health data. Our proposed framework emphasises techniques that better match the unique demands of a fog environment. In particular, our unique framework meets the following key research gaps for an effective remote patient monitoring in healthcare services:

- The importance of a privacy-centric approach in the design of fog environments. An existing study [35] emphasises the imperative of proactive privacy practices given that fog nodes live close to sensitive data sources. Trust-based and privacy-protected frameworks combined with encryption of sensitive information such as identity, authentication, location and other attributes associated with each fog node would establish reliable communication across distributed healthcare networks [36,37].
- The use of lightweight encryption techniques, which a fog environment necessitates, has given the limited availability of computational resources. For example, data masking and anonymisation, where the data is needed only for analysis rather than for complete retrieval. Additionally, determining the level of Homomorphic Encryption —partial (PHE) or full (FHE) —involves a trade-off between performance and security.
- The limitations of battery life in IoT devices emphasise the importance of this trade-off. In our smart remote patient monitoring case study, the successful use of lightweight techniques was demonstrated by maintaining patient health data confidentiality during transmission by low-power wearable devices.
- Considerations for fog-optimised data management, particularly database optimisation and the efficiency of query strategies. Non-relational databases can play a vital role in fog computing by offering scalability and flexibility. Relational databases, for instance, may experience latency when complex joins are required across fog nodes. Besides the performance and uptime issues faced by relational databases, they also pose risks to data security and privacy [38].

### 5.2. Limitations of the Study and Future Recommendations

This research limits its focus on uniquely integrating IDP, security mechanisms and novel AI-based anomaly detection over fog computing environment for a secure data transmission in supporting critical healthcare scenarios. We acknowledge that this proof-of-concept development and experimental validation of our framework is limited to certain enhancements in the existing literature and there are several future research possibilities that could emerge from this work. We have evolved the traditional RF AI strategies by pushing its boundaries in a unique way of enhancing the algorithm with intelligent data prioritisation for a dynamically generated high volume clinical data within a fog environment. In addition, the security of these high-priority health data were continuously observed by machine learning models trained to detect anomalies and threats in real time. Overall, in each data processing tier of our proposed framework, we have enhanced the existing models adopting the best approaches within the simulated environment of the study. Researchers should further analyse how these tiers can further be optimised and integrated. Thought can be given to:

- Analyse workload characteristics of CPS applications (data volume, processing intensity, real-time requirements) to tailor hybrid architectures. Existing work [35,39] on workload modelling for CPS in hybrid environments has shown that understanding factors such as data volume, processing intensity, and real-time requirements is crucial for tailoring the hybrid architecture appropriately.
- Develop automated workload scheduling tools for efficient tasks and resource distribution across cloud, fog, and edge tiers.
- Apply several other AI and deep learning algorithms, such as PCA, Autoencoders-DNN, RNN, GNN, GAN, LSTM, etc., not only for bottleneck detection but also for anomaly detection and strengthening security in prioritised health data streams [40].
- Explore collaboration between fog nodes (load balancing, failover, resource sharing) to improve resilience and scalability.
- The use of multi-factor authentication. For example, applying a zero-trust model (which can be particularly important in fog environments where different manufacturers produce devices and may run other operating systems) and exploring continuous authentication techniques, such as keystroke analysis and device usage patterns, can be beneficial.

There are other proactive monitoring techniques specific to fog nodes and CPS vulnerabilities that this paper does not discuss. Rapidly developing AI tools will open new possibilities and pose new threats. Our case study demonstrated the value of machine learning algorithms, which successfully detected anomalous device usage patterns and helped secure against data breaches. Other examples are the use of blockchains for integrity and approaches to fog node hardening that pay greater attention to the disparate hardware and software employed across fog environments [10,41].

## 6. Conclusions

This study explored the potential of fog computing to address latency, bandwidth, and security issues in IoT-enabled CPS. We introduced a secure fog-based framework that incorporates lightweight encryption, an IDP, and AI-based anomaly detection. In a simulated remote health monitoring scenario, the framework successfully prioritised critical clinical data, accurately detected anomalies by formulating an AI-based enhanced Random Forest model, and maintained privacy despite resource constraints. These findings from the simulated experiment verify the feasibility of the framework and the simulated experiment in enabling proactive interventions, reducing dependency on centralised cloud infrastructure, and improving patient outcomes in remote healthcare. Although demonstrated in healthcare, the framework can be applied to other CPS fields such as autonomous vehicles, smart cities, and energy systems. Overall, this work presented a scalable, privacy-preserving architecture that combines secure communication, real-time data prioritisation, and robust AI-based anomaly and threat detection with utmost reliability to strengthen fog computing applications in sensitive, distributed settings.

This study contributes to the IS and healthcare domains by integrating fog computing, security (IDP), and AI-based anomaly detection into a unified framework which advances intelligent data processing studies further. Future work will involve deploying the framework in real healthcare environments to test its robustness and clinical usefulness, as the findings of this study are initial since they have not yet been validated with real-world datasets. Additionally, future efforts will expand this study to incorporate advanced deep learning algorithms and other AI techniques for analysing prioritised health data and detecting anomalies early using the simulated dataset.

## Figures and Tables

**Table 1:** Comparison among Cloud, Fog, and Edge Computing.

Feature	Edge Computing	Fog Computing	Cloud Computing
**Processing** **Location**	On or near the data-generating devices (sensors, gateways)	Intermediate layer between edge and cloud (local servers, routers)	Centralised data centres located far from end devices
**Latency**	Very low (milliseconds)	Low (near real-time)	High (dependent on network distance)
**Scope of** **Processing**	Device-level processing	Regional/local aggregation of multiple edge devices	Global-level processing and storage
**Scalability**	Limited to device capacity	Moderate—scalable within local networks	Highly scalable but bandwidth dependent
**Security and Privacy**	Basic device-level security	Enhanced localised security and privacy control	Centralised security; higher risk during data transmission
**Real-time** **Analytics**	Fast for simple tasks	Suitable for time-sensitive, distributed analytics	Limited for latency-sensitive operations
**Example** **Applications**	Smart vehicles, industrial sensors	Smart healthcare, intelligent transport, smart grids	Data storage, deep analytics, and training of AI models

**Table 2:** Description of attributes of the Simulated Dataset.

Attribute	Description
**Device ID**	Unique identifier for each Fitbit device (1 to 5).
**Timestamp**	Hourly timestamp for each day in one month.
**Heart Rate**	Simulated heart rate values (60–180 bpm).
**Heart Rate Priority**	Priority classification of heart rate values (high) based on critical readings exceeding 100 bpm.
**SpO_2_**	Simulated oxygen saturation levels in the blood.
**SpO_2_ Priority**	Priority classification of SpO_2_ values (high/low), e.g., values below 90% flagged as urgent.
**Breathing Rate**	Simulated breathing rate values.
**Breathing Rate Priority**	Priority classification of breathing rate values (high/low), e.g., less than 12 or greater than 20 breaths per minute, is flagged as urgent.
**Exercising**	Indicator of whether the individual is exercising (Yes/No).
**Skin Temperature**	Simulated skin temperature values.
**Heart Rate Variability**	Simulated heart rate variability values to capture variability in cardiac activity.
**Resting Heart Rate**	Simulated resting heart rate values.
**Status**	A label (“ok” or “alert”) derived from combined thresholds, used for AI-based anomaly detection.

**Table 3:** Key performance metrics of the model.

Metric	Formula	Calculation	Result
**Accuracy**	(TP + TN)/(TP + TN + FP + FN)	(3500 + 5695)/10,000 = 9195/10,000	0.935 → 93.5%
**Precision**	TP/(TP + FP)	3500/(3500 + 355) = 3500/3855	0.908 → 90.8%
**Recall**	TP/(TP + FN)	3500/(3500 + 450) = 3500/3950	0.887 → 88.7%
**F1-Score**	2 × (Precision × Recall)/(Precision + Recall)	2 × (0.908 × 0.887)/(0.908 + 0.887) = 2 × 0.805/1.795	0.897 → 89.7%

## Data Availability

The data presented in this study are available on request from the corresponding author. The data are not publicly available due to privacy and ethical restrictions of health data regulations.

## Abbreviations

The following abbreviations are used in this manuscript:

AI	Artificial Intelligence
CPS	Cyber–Physical System
IoT	Internet of Things
IDP	Intelligent Data Prioritisation
DPE	Data Protection Engine
AES	Advanced Encryption Standard
PG	Privacy Gateway
IAM	Identity and Access Management
IDS	On-Node Intrusion-Detection System
SpO_2_	Oxygen Saturation
PHE	Partial Homomorphic Encryption
FHE	Full Homomorphic Encryption
